# The Immune Modulation HLA-G*01:01:01 Full Allele Is Associated with Gastric Adenocarcinoma Development

**DOI:** 10.3390/ijms251910645

**Published:** 2024-10-03

**Authors:** Fabio Suarez-Trujillo, Ignacio Juarez, Christian Vaquero-Yuste, Alberto Gutierrez-Calvo, Adela Lopez-García, Inmaculada Lasa, Remedios Gomez, José Manuel Martin-Villa, Antonio Arnaiz-Villena

**Affiliations:** 1 Department of Immunology, School of Medicine, Complutense University of Madrid, 28040 Madrid, Spain; fabiosuareztr@hotmail.com (F.S.-T.); ignajua@ucm.es (I.J.); chvaq01@ucm.es (C.V.-Y.); jmmvilla@ucm.es (J.M.M.-V.); 2 Digestive and General Surgery Service, Principe de Asturias University Hospital, 28015 Madrid, Spain; agutierrezcalvo@telefonica.net (A.G.-C.); adelapetra.lopez@salud.madrid.org (A.L.-G.); inmaculada.lasa@salud.madrid.org (I.L.); remedios.gomez@salud.madrid.org (R.G.)

**Keywords:** HLA, *HLA-G*, non-classical HLA, cancer, gastric adenocarcinoma, HLA-G*01:01:01, complotypes, haplotypes, immune modulation, tolerance

## Abstract

The Human Leukocyte Antigen (HLA) system contains a set of genes involved at many levels in the innate and adaptive immune response. Among the non-classical HLA class I genes, HLA-G stands out for the numerous studies about its pivotal role in regulating/modulating immune responses. Also, its involvement in extravillous cytotrophoblast function, viral infections, autoimmunity, and cancer has been extensively documented. The present study explores for the first time the relationship between natural alleles of HLA-G, rather than STSs, SNPs, or partial gene polymorphisms, and the development of gastric adenocarcinoma, by analyzing the genetic profile of a cohort of 40 Spanish patients with this type of tumor using DNA extracted from paired biopsies of tumoral and adjacent non-tumoral gastric tissue. Our results reveal a significant statistical relationship between the presence of the HLA-G*01:01:01 allele and the development of gastric cancer, while other common alleles such as -G*01:04 or -G*01:05N did not demonstrate a significant correlation. Studying the involvement of *HLA* genes in the development of many diseases is relevant to understanding their pathophysiology. However, the absence of specific mechanisms underlying these associations suggests that investigating complete HLA natural alleles’ extended haplotypes or complotypes may offer a more precise and valuable approach to elucidating the association of HLA with the pathogenesis of disease.

## 1. Introduction

**Figure 1:**
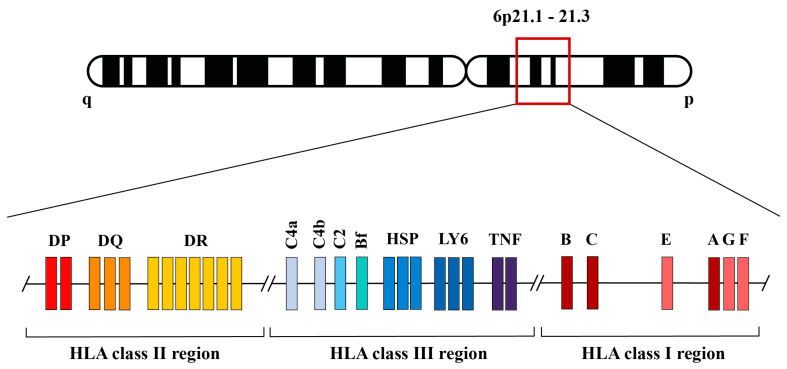
Representation of the HLA genomic region situated on the human chromosome 6 6p21.1-21.3 band. Non-classical class I HLA genes are located in the telomeric part of HLA region [1,2].

The Human Leukocyte Antigen (HLA) comprises a set of genes located in human chromosome 6 which codifies for key molecules in the adaptive and innate immune responses [1,2]. Class I, II, and III genes are clustered here including class I and II HLA genes for antigen presentation, complement genes (such as Factor B, C2, or C4) and other important genes like vomeronasal receptors, chaperons, or lymphocyte antigens (Figure 1) [1,2]. Classical class I (*HLA-A*, *-B*, *-C*) and class II (*HLA-DR*, *-DQ*, *-DP*) genes encode for molecules that present antigen peptides to clonotypic T-cell receptors on the surface of CD8 + cells and CD4 + cells, respectively. Non-classical class I proteins (HLA-G, -E, -F) have been implicated in modulating immune system activity [3,4,5].

**Figure 2:**
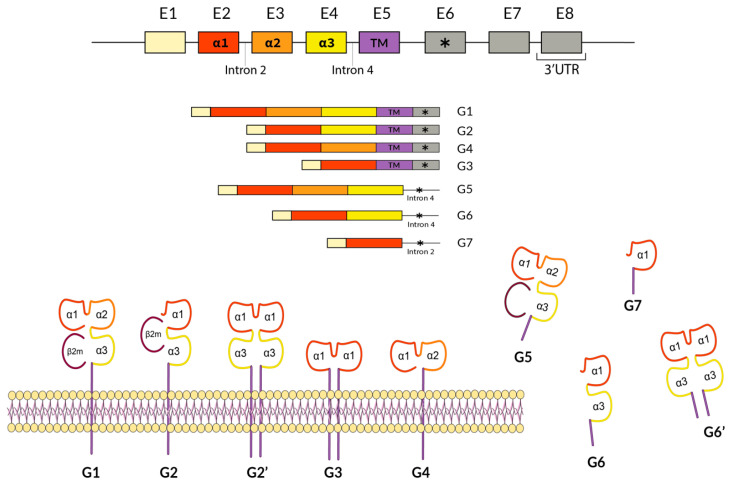
Genetic structure of the *HLA-G* gene, showing transcription and translation of the seven isoforms described. Note that some functional isoforms lack most of the α domain but are still functional, even with only 1 domain (G3, G7) [7]. * symbol denotes a STOP codon. A more detailed introduction to *HLA-G* alleles structure and function is found in [6,7].

The HLA-G protein is composed of a heavy chain displaying α1, α2, and α3 domains, which is bound to β-2 microglobulin acting as a light chain (Figure 2) [6,7]. The *HLA-G* gene comprises eight exons that encode the domains present in the heavy chain. Exons 2, 3, and 4 encode for α1, α2, and α3 extracellular domains, with the α1 and α2 domains responsible for the antigen presenting a valve-like structure (Figure 2) [6,7]. Exons 5 and 6, respectively, codify for the transmembrane region and the cytoplasmic domain [6,7,8]. Exon 7 is transcribed in the pre-mRNA molecule but is absent in mature mRNA, and exon 8 is not translated but bears the 3′UTR region crucial for regulating HLA-G expression [5,6]. Seven different isoforms have been described for HLA-G (Figure 2); some of them lack certain structural extracellular domains yet remain functional (see HLA-G3 and G7 structures in Figure 2) [5,6,7].

As a non-classical HLA class I gene, *HLA-G* shows restricted expression in the organism, being mainly present in the extravillous cytotrophoblast cells, cornea, proximal nail matrix, thymus, hematopoietic stem cells, and pancreas [9,10,11,12,13,14,15], while HLA classical class I molecules are expressed in all nucleated cells. HLA-G is involved in a wide variety of processes, due to its tolerogenic functions such as maternal immune acceptance of the foetus [16], organ transplantation [16], viral infections [17,18], autoimmunity [17,19], and cancer progression [20].

Gastric epithelial adenocarcinomas represent 90% of the stomach tumors currently diagnosed and are usually located in the cardia (31.0%) or antrum (26.0%). Many studies have correlated the presence of *Helicobacter pylori* with development of gastric adenocarcinomas. This bacterium is present in 84% of gastric adenocarcinoma-diagnosed patients [21]. By 2022, stomach cancer was the fifth most frequent cancer diagnosed worldwide (970,000 cases, 4.9% of all newly diagnosed cases of cancer), also ranking in the fifth position in terms of mortality (6.8% of cancer deaths in the world) [22]. The 5-year overall age-standardized relative survival rate in the 2017–2021 period was 42.9%, slightly increased in comparison with previous intervals [23].

Stomach adenocarcinomas are usually targeted by the immune system due to their high expression of neo-antigens, similar to other tumors with high frequencies of somatic mutations. However, the expression of immunomodulatory molecules such as HLA-G by tumoral cells enables cancer to evade immune surveillance and the effector responses of the immune system against it. HLA-G plays a key role in the cancer immunoediting mechanism, attenuating the elimination of tumoral cells [20,24,25,26].

In the present study, we studied the polymorphism of *HLA-G* genes in a cohort of Spanish gastric adenocarcinoma patients, to determine whether variations in the *HLA-G* gene were associated with genetic risks or protective factors influencing cancer progression and survival.

## 2. Results

### 2.1. HLA-G Allelic Distribution in Spanish Gastric Adenocarcinoma Patients

This is the first study that has reported the HLA-G allelic profile of Spanish patients with gastric adenocarcinoma. In the present paper, we compare the HLA-G profile of our gastric adenocarcinoma cohort with that of a healthy Spanish cohort previously published [27] (Table 1). The HLA-G alleles expressed in the patients studied in this work were as follows: HLA-G*01:01:01 (80%), -G*01:01:08 (5%), -G*01:04:01/*01:23* (5%), -G*01:05N (7.5%), and -G*01:06:01 (2.5%) (Table 2).

### 2.2. Allele Ambiguities Are Found in Tumoral Tissue Samples but Not in Paired Non-Tumoral (Distal) Tissue Samples

Regarding *HLA-G* typed alleles, discordances were observed in 3 out of 40 sequenced patients between the typed allele in the tumoral tissue sample and its distal (non-tumoral) tissue paired sample (Table 3). Patients 18 and 30 expressed HLA-G*01:01:08 in their healthy tissue, while the HLA-G expression in tumor tissue was unclear and the typing ambiguities led to potential assignments of alleles such as *01:01:01*, *01:01:06*, *01:01:08*, or *01:03:01* (patient 18), and *01:01:02*, *01:01:22*, *01:24*, or *01:26* (patient 30). Similarly, patient 17 showed HLA-G*01:06:01 in the healthy tissue, with ambiguous tumoral tissue typing potentially matching HLA-G*01:01:02, *01:01:22*, *01:24*, or *01:26* alleles.

### 2.3. Statistical Comparisons within the Gastric Cancer Cohort: Significance of Tumoral Ambiguities Found

Fisher’s exact test (FET) was applied to evaluate the relationship between typing ambiguities and sample type (tumoral or non-tumoral) within our gastric cancer cohort. The statistical test showed a non-significant *p*-value of 0.24. This suggested that the presence of ambiguities during the *HLA-G* typing was not associated with the sample type in our gastric cancer group. However, the reduced size of our sample may have influenced the results and further investigations with a larger cohort are needed in order to clarify these findings.

### 2.4. Statistical Comparisons between the Gastric Cancer Spanish Cohort (n = 40) and the Healthy Spanish Cohort (n = 114)

#### 2.4.1. Fisher’s Exact Text and Odds Ratios Calculations for Common Alleles in Both Cohorts (HLA-G*01:01:01, HLA-G*01:04 and HLA-G*01:05N)

**Figure 3:**
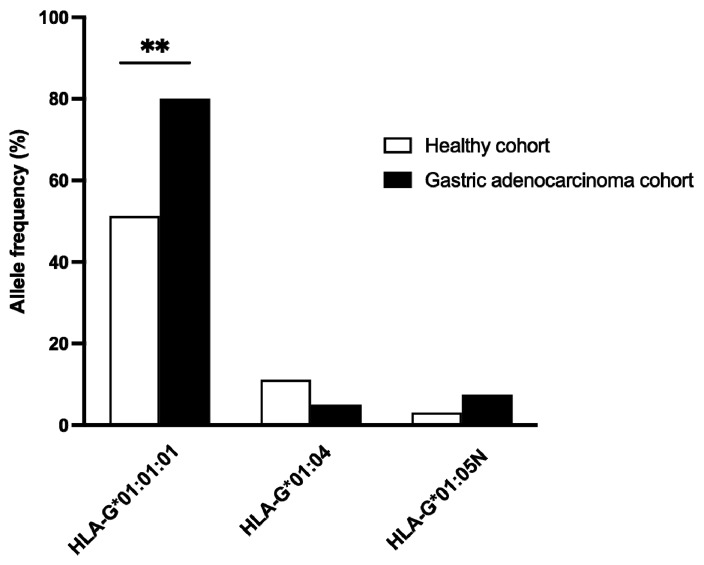
Frequencies (%) of each common allele in gastric cancer (40) and healthy (114) cohorts. HLA-G*01:01:01 was the only allele significantly overrepresented (based on Fisher’s exact test; *p*-value = 0.0014) in gastric cancer individuals vs the healthy cohort. ** indicates a significant association with *p* below 0.01.

Among the common alleles examined (Figure 3), HLA-G*01:01:01 was identified as a significant genetic factor associated with gastric cancer. The Fisher’s exact test revealed a remarkably low *p*-value of 0.0014, strongly indicating a significant association of HLA-G*01:01:01 with gastric cancer. An odds ratio of 3.83 also supported this association. Conversely, no statistically significant correlation was found between the HLA-G*01:04 and HLA-G*01:05N alleles and gastric adenocarcinoma, with *p*-values of 0.3564 and 0.3768, respectively. These findings suggest that HLA-G*01:01:01 may contribute to susceptibility to gastric cancer, underscoring the need for a comprehensive longer cohort study to validate this genetic relationship.

#### 2.4.2. Principal Component Analysis (PCA) for Each Allele’s Contribution to Gastric Adenocarcinoma Development

**Figure 4:**
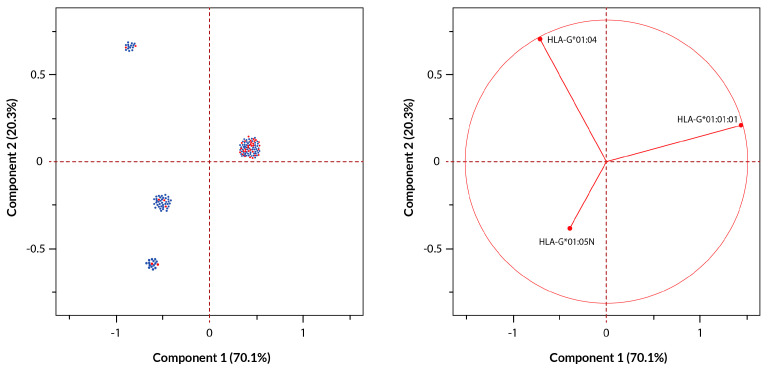
Principal component analysis (PCA) of the HLA-G*01:01:01, HLA-G*01:04, and HLA-G*01:05N frequencies in healthy individuals and our gastric adenocarcinoma cohort. The left graph shows the spatial distribution of individuals of both cohorts along PC1 and PC2, which explain 70.1% and 20.3% of the variance, respectively. Healthy individuals are represented with blue dots and gastric adenocarcinoma individuals are represented with red dots. The biplot graph on the right shows the contribution of each studied allele to the variance represented by PC1 and PC2. HLA-G*01:01:01 is strongly linked to the cluster of gastric adenocarcinoma individuals, showing correlation between this allele and cancer. Overlapped points are shown as swarms for better visualization.

Principal component analysis was also carried out, focusing solely on the *HLA-G* alleles shared by both cohorts (HLA-G*01:01:01, HLA-G*01:04, and HLA-G*01:05N). The PCA results indicated that Principal Component 1 (PC1) and Principal Component 2 (PC2) together accounted for the majority of the variance among individuals, explaining 70.1% and 20.3% of the variance, respectively, clearly separating most of the gastric adenocarcinoma individuals carrying the HLA-G*01:01:01 allele from those carrying other alleles. Results of the PCA are depicted in Figure 4, where blue dots represent healthy individuals and red dots represent gastric adenocarcinoma individuals. PC1 and PC2 are plotted on the X and Y axes of the graph, respectively, demonstrating that the majority of cancer patients carrying the HLA-G*01:01:01 allele were cluster together in the positive halves of both axes. This clustering indicates a strong correlation between the presence of the HLA-G*01:01:01 allele and the development of gastric adenocarcinoma.

## 3. Discussion

### 3.1. HLA-G Frequencies and Polymorphism in the Spanish Gastric Adenocarcinoma Cohort Studied

HLA-G polymorphism has scarcely been studied previously in the Spanish population. The data obtained in the present study were compared with HLA-G polymorphism data from healthy Spanish controls previously published in [27]. In this case, and since only exons 2, 3, and 4 were sequenced, *HLA*-G*01:04:01 and HLA-G*01:23 alleles were indistinguishable at the time of typing as they had the same sequence in these exons. HLA-G*01:23 is an allelic variant arising from the HLA-G*01:04:01 allele, and they only differ in a C→T nucleotide change at position +37 of the allelic sequence (leader peptide) [28]. Thus, these patients (4 and 38) may probably have been *HLA*--G*01:04:01 expressors, because the *HLA*-G*01:23 allele has been found only once in a Norwegian population sample and has not been confirmed in other populations [28], and -G*01:04:01 is a more frequent allele and has been confirmed by different laboratories around the world. In this study, HLA-G*01:04:01 was considered as HLA-G*01:04 for statistical comparisons.

### 3.2. Contribution of HLA-G*01:01:01 Allele to Gastric Cancer

Among the common alleles investigated in both the gastric cancer and healthy cohorts, HLA-G*01:01:01 emerged as a significant genetic factor strongly associated with gastric cancer (*p*-value of 0.0014 in Fisher’s exact test). On the other hand, the odds ratio of 3.83 in the FET indicated that individuals bearing HLA-G*01:01:01 were almost four times as likely to have gastric cancer compared within dividuals without this allele. The combination of a low *p*-value and a high odds ratio confirms a statistically significant association between HLA-G*01:01:01 and gastric cancer. Further research is needed comparing a larger cohort to assess the role of HLA-G*01:01:01 in gastric cancer development and progression. In contrast, HLA-G*01:04 and HLA-G*01:05N did not show significant associations with gastric cancer susceptibility in our study (*p*-value = 0.3869 and *p*-value = 0.5475, respectively). The results from the Fisher’s exact test and Chi-squared test provide robust statistical evidence of these non-significant associations.

Our principal component analysis (PCA) revealed that HLA-G*01:01:01 exhibited a strong correlation with PC1 and PC2, again suggesting a potential role in gastric cancer susceptibility. Conversely, HLA-G*01:04 and HLA-G*01:05N had more variable and non-significant relationships with these principal components, indicating the lower contribution of these alleles to the disease.

Taking all these findings together, we put forward a clear association between the presence of HLA-G*01:01:01 and the development of gastric adenocarcinoma. However, further studies are necessary to investigate the association of other HLA-G alleles with gastric cancer, in terms of both risk and protection. Previous studies have also put forward the role of the HLA-G*01:01 allelic group in cancer susceptibility. This is the case for bladder transitional cell carcinoma, which was associated with the presence of HLA-G*01:01 in a Brazilian cohort [29]. On the other hand, HLA-G*01:01:01 was not associated with gastric adenocarcinoma in an Iranian cohort, but other HLA-G*01:01 family alleles, such as HLA-G*01:01:02 (risk), HLA-G*01:01:03 (protective), and HLA-G*01:01:08 (protective) showed association with this type of gastric tumor [30].

This new association of HLA-G*01:01:01 with gastric cancer and its potential use as a predictive biomarker of disease should be taken into consideration together with other classical gastric cancer predictive markers such as CEA and CA-19-9, commonly used in clinical practice [31]. The use of these disease biomarkers in conjunction with genetic screening for HLA-G*01:01:01 could provide greater robustness in the early prediction of gastric cancer. HLA-G*01:01:01 could be used as a marker for early warning of the disease and combined with classical biomarkers to achieve a more effective and efficient diagnosis.

### 3.3. Importance of Haplotypes/Complotypes for HLA-Disease Associations Instead of Single-Allele Studies

Adaptive (classical class I and class II HLA), innate (C2, C4, Bf), and modulatory (non-classical HLA class I) immunity genes have been evolutionarily maintained as a block to be inherited together. This characteristic makes the study of haplotypes and complotypes more useful and logical for associating HLA with diseases. This approach was first proposed by Roger Dawkins’ group in the late 1980s when they found haplotypes and complotypes associated with different diseases such as systemic lupus erythematosus, myasthenia gravis, or rheumatoid arthritis, among others [32,33]. Since then, various diseases have also been associated with extended HLA haplotypes, such as type I diabetes [34], celiac disease [35], or psoriatic arthritis [36].

The results of this study are significant as this is the first instance where the HLA allelic profile of a non-classical gene, crucial in cancer immune evasion, has been associated with HLA-G in a cohort of Spanish gastric cancer patients. However, the conclusions presented should be interpreted with caution due to the limited sample size. Ongoing research aims to acquire the extended haplotype profiles of these non-classical HLA genes, which will enhance the overall data and facilitate the establishment of a more robust statistical correlation with the disease.

### 3.4. Full Alleles in HLA-Disease Associations

The value of studying full HLA alleles associated with diseases instead of other polymorphisms that are associated with full alleles is obvious to us [6,7]. It has already been stated that during the past 50 years, small pathogenetic advances have been firmly established through studying even natural single full alleles [6,7]. Conjoint HLA alleles from different loci or extended HLA haplotypes should be studied to try to overcome this lack of a universal and sound explanation regarding HLA and disease pathogenesis. At present, a new epoch is starting to repeat the HLA single-allele studies with non-classical class I HLA immunomodulatory alleles. This may still not be enough, although it may be more promising for autoimmune diseases and also tumors. Our view is that studying not full non-classical class I alleles (more difficult to detect at present), but linked polymorphisms instead, will continue to worsen the expectations that HLA alleles indirectly linked to these easier-to-detect polymorphisms are going to give an answer to the question of HLA and disease physiopathology. HLA physiology may still not be fully uncovered, but it is at least necessary to directly relate alleles’ function with physiopathology [6,7]. A sign that HLA physiology is not fully understood is that transplantation matching effects and HLA/disease association have been related without a firm basis to the putative single function of HLA molecules as antigen presenters [3,5]. Thus, another mistake is to continue with indirect measurement of non-classical class I HLA alleles and diseases with different markers [37,38,39] that in turn are associated with true HLA alleles. Finally, the present study should be expanded; non-classical class I HLA full-allele research in relation to disease should be common and not an exception.

## 4. Materials and Methods

### 4.1. Samples

A total of 40 unrelated patients diagnosed with gastric adenocarcinoma were included in this study. Patients were classified according to the TNM staging criteria (stages I through IV) [40]. The inclusion criteria for this study were patients (women or men) over 18 years old with gastric adenocarcinoma stratified according to UICC/AJCC criteria (seventh edition 2009) [40] at any TNM stage and with Karnofsky index >70% or performance status ≤2. Tumoral tissue and distal (non-tumoral) tissue were taken from each patient, finally having two tissue samples from each patient participating in the study. All patients signed to provide their informed consent before inclusion in the study, and all samples were anonymized upon arrival at the laboratory. The exclusion criteria included non-resecability of the primary tumor, coexistence with other neoplastic diseases, pregnancy, and severe alterations of hepatic, cardiovascular, or renal function.

### 4.2. DNA Extraction, Amplification and Sequencing

Genomic DNA from tumoral/adjacent tissue was extracted using the Nucleon™ PhytoPure™ Genomic DNA Extraction Kit (Cytiva, Marlborough, MA, USA; ref. RPN8501) following the protocol provided by the manufacturer. DNA amplification of exons 2, 3, and 4 of HLA-G was carried out separately by direct PCR. The sequences of the different HLA-G exons were obtained using the following specific 5′ and 3′ primers: exon 2: forward: 5′-GAGGGTCGGGCGGGTCTCAAC-3′, reverse: 5′-GCATGGAGGTGGGGGTCGTGA-3′; exon 3: forward 5′-TGGGCGGGGCTGACCGAGAAGGTGG-3′, reverse: 5′-CTCTCCTTGTGCTAGGCCAGGCTGAGA-3′; exon 4: forward: 5′-CCATGAGAGATGCAAAGTGCT-3′, reverse: 5′-TGCTTTCCCTAACAGACATGA-3′. The PCR conditions consisted of 1 cycle of 95 °C, 15 min; 95 °C, 0.5 min; 35 cycles of 68 °C, 0.5 min; 72 °C, 0.67 min; and 1 cycle of 72 °C, 10 min for exon 2; 1 cycle of 94 °C, 2 min; 94 °C, 1 min; 35 cycles of 67 °C, 1.5 min; 72 °C, 2 min; and 1 cycle of 72 °C, 10 min for exon 3; and 1 cycle of 95 °C, 5 min; 95 °C, 0.67 min; 35 cycles of 58 °C, 0.67 min; 72 °C, 0.67 min; and 1 cycle of 72 °C, 10 min for exon 4, in a programmable thermocycler (Mastercycler Epgradient S, Eppendorf, Hamburg, Germany). The products obtained were electrophoresed in 2% agarose gel and detected by staining with Midori Green dye (Nippon Genetics Europe, Düren, Germany; ref. MG04) and were purified using the Mini Elute Gel Extraction Kit 250 (Qiagen, Hilden, Germany; ref. 28604). Purified products were sequenced in both directions using an ABI 3730 DNA analyzer (Applied Biosystems, Foster City, CA, USA) using the same primers as the amplification process. *HLA-G* polymorphism was identified by aligning the sequences with already identified alleles [41] using Mega 7 software [42].

### 4.3. Statistical Analyses

Statistical tests were performed using R language and a *p*-value < 0.05 was considered for statistical significance in all tests applied in this work. Fisher’s exact test was applied within the gastric cancer cohort in order to assess linkage between the presence of ambiguities in *HLA-G* typing and the sample type (tumoral or non-tumoral); this method is commonly employed in reduced-size samples. At the time of typing, 37 out of 40 individuals exhibited a clear *HLA-G* allele in both tumoral and non-tumoral samples. Meanwhile, *HLA-G* allele frequencies obtained in our gastric adenocarcinoma cohort were statistically compared with those of a healthy Spanish cohort previously published (Table 1), by applying Fisher’s exact test. Furthermore, JMP v17.2 software (SAS Institute Inc., Cary, NC, USA) (https://www.jmp.com/en_us/home.html; accessed on 10 June 2024) was employed for performing principal component analysis using *HLA-G* alleles found in common in both cohorts, in order to better obtain visualization of the *HLA-G* alleles’ contribution to gastric adenocarcinoma status. Graphs were constructed with GraphPad Prism software v.9.3.1 and Adobe Illustrator 2020.

## 5. Conclusions

After the evaluation of the *HLA-G* genetic profile of individuals with gastric adenocarcinoma included in present work in comparison with the profile of a previously studied healthy cohort, it is concluded that the presence of the HLA-G*01:01:01 allele is a risk factor for the development of this type of tumor. Other alleles such as HLA-G*01:04 or HLA-G*01:05N do not show a significant statistical correlation with the disease. The role of HLA-G*01:01:01 may have considerable weight in the development of this pathology, but it has to be interpreted in conjunction with other alleles of the HLA system (haplotypes) that are inherited together as a block; the study of haplotypes or complotypes together and an additional bigger cohort will provide more information and will allow researchers to establish more robust statistical correlations between HLA and disease. Our group is carrying out more studies to address these issues. We also conclude the following:

(1) The long-lasting negative results in relation to the HLA–disease pathogenesis linkage are probably a reflection of a fundamental lack of knowledge of full HLA/MHC (major histocompatibility complex) function (physiology). It may involve not only antigen presentation or a cluster of HLA/MHC genes that work together for microbial and self-defense, and other undetermined factors like microbiota could also be important [6,7,43];
(2) A further uncertainty is added by not studying full alleles directly, as is generally the case (and sometimes confirmatory [44]) when using class I non-classical modulatory genes, because of more difficult typing. These are usually detected by indirect related markers [37,38,39]. Widespread use of full MHC alleles should be practiced instead in order to minimize the lack of pathogenesis explanation results from HLA/disease studies in the past 50 years.

## Figures and Tables

**Table 1:** Comparison between HLA-G allele frequencies found in a healthy Spanish population sample (*n* = 114) [27] and the Spanish gastric adenocarcinoma patients studied in present work (*n* = 40).A more complete introduction to HLA-G alleles is found in [6,7].

Healthy Population (*n* = 114)		Gastric Adenocarcinoma Population (*n* = 40)	
*HLA-G* Allele	Frequency (%)	*HLA-G* Allele	Frequency (%)
*01:01:01*	51.31	*01:01:01*	80.00
*01:01:02*	25.10	*01:01:08*	5.00
*01:01:03*	7.26	*01:04:01/01:23*	5.00
*01:04*	11.11	*01:05N*	7.5
*01:05N*	3.09	*01:06:01*	2.50

**Table 2:** HLA-G allele frequencies found inthe Spanish gastric adenocarcinoma patients included in present work (*n* = 40).

Patient	Sample (T/NT)	*HLA-G* Allele
1	Tumoral	*01:05N*
	Non-tumoral (distal)	*01:05N*
2	Tumoral	*01:01:01*
	Non-tumoral (distal)	*01:01:01*
3	Tumoral	*01:01:01*
	Non-tumoral (distal)	*01:01:01*
4	Tumoral	*01:04:01/01:23*
	Non-tumoral (distal)	*01:04:01/01:23*
5	Tumoral	*01:01:01*
	Non-tumoral (distal)	*01:01:01*
6	Tumoral	*01:01:01*
	Non-tumoral (distal)	*01:01:01*
7	Tumoral	*01:01:01*
	Non-tumoral (distal)	*01:01:01*
8	Tumoral	*01:01:01*
	Non-tumoral (distal)	*01:01:01*
9	Tumoral	*01:01:01*
	Non-tumoral (distal)	*01:01:01*
10	Tumoral	*01:01:01*
	Non-tumoral (distal)	*01:01:01*
11	Tumoral	*01:01:01*
	Non-tumoral (distal)	*01:01:01*
12	Tumoral	*01:01:01*
	Non-tumoral (distal)	*01:01:01*
13	Tumoral	*01:01:01*
	Non-tumoral (distal)	*01:01:01*
14	Tumoral	*01:01:01*
	Non-tumoral (distal)	*01:01:01*
15	Tumoral	*01:01:01*
	Non-tumoral (distal)	*01:01:01*
16	Tumoral	*01:01:01*
	Non-tumoral (distal)	*01:01:01*
17	Tumoral	*01:01:02/01:01:22/01:24/01:26*
	Non-tumoral (distal)	*01:06:01*
18	Tumoral	*01:01:08/01:01:06/01:03:01/01:01:01*
	Non-tumoral (distal)	*01:01:08*
19	Tumoral	*01:01:01*
	Non-tumoral (distal)	*01:01:01*
20	Tumoral	*01:01:01*
	Non-tumoral (distal)	*01:01:01*
21	Tumoral	*01:05N*
	Non-tumoral (distal)	*01:05N*
22	Tumoral	*01:01:01*
	Non-tumoral (distal)	*01:01:01*
23	Tumoral	*01:01:01*
	Non-tumoral (distal)	*01:01:01*
24	Tumoral	*01:01:01*
	Non-tumoral (distal)	*01:01:01*
25	Tumoral	*01:01:01*
	Non-tumoral (distal)	*01:01:01*
26	Tumoral	*01:01:01*
	Non-tumoral (distal)	*01:01:01*
27	Tumoral	*01:01:01*
	Non-tumoral (distal)	*01:01:01*
28	Tumoral	*01:01:01*
	Non-tumoral (distal)	*01:01:01*
29	Tumoral	*01:05N*
	Non-tumoral (distal)	*01:05N*
30	Tumoral	*01:01:02/01:01:22/01:24/01:26*
	Non-tumoral (distal)	*01:01:08*
31	Tumoral	*01:01:01*
	Non-tumoral (distal)	*01:01:01*
32	Tumoral	*01:01:01*
	Non-tumoral (distal)	*01:01:01*
33	Tumoral	*01:01:01*
	Non-tumoral (distal)	*01:01:01*
34	Tumoral	*01:01:01*
	Non-tumoral (distal)	*01:01:01*
35	Tumoral	*01:01:01*
	Non-tumoral (distal)	*01:01:01*
36	Tumoral	*01:01:01*
	Non-tumoral (distal)	*01:01:01*
37	Tumoral	*01:01:01*
	Non-tumoral (distal)	*01:01:01*
38	Tumoral	*01:04:01 / 01:23*
	Non-tumoral (distal)	*01:04:01 / 01:23*
39	Tumoral	*01:01:01*
	Non-tumoral (distal)	*01:01:01*
40	Tumoral	*01:01:01*
	Non-tumoral (distal)	*01:01:01*

**Table 3:** Patients exhibited HLA-G typing ambiguities solely in the tumoral tissue sample. *HLA-G* alleles were correctly detected in non tumoral (distal) samples.

Patient	Sample (T/NT)	HLA-G Allele
17	Tumoral	*01:01:02/01:01:22/01:24/01:26*
	Non-tumoral (distal)	*01:06:01*
18	Tumoral	*01:01:01/01:01:06/01:01:08/01:03:01*
	Non-tumoral (distal)	*01:01:08*
30	Tumoral	*01:01:02/01:01:22/01:24/01:26*
	Non-tumoral (distal)	*01:01:08*

## Data Availability

The genetic profile of individuals analyzed in present work is publicly available in Table 2 of this paper and Universidad Complutense de Madrid, Immunology Department repository.
